# A human-on-human assay for detecting anti-myocardial antibodies in patients with myocardial disease

**DOI:** 10.3389/fimmu.2026.1744039

**Published:** 2026-05-08

**Authors:** Anne Auer, Johanna Siegel, Stasa Janjatovic, Margarete Heinrichs, Stefan Störk, Peter Heuschmann, Caroline Morbach, Katrin Streckfuss-Bömeke, Gustavo C. Ramos, Katrin G. Heinze

**Affiliations:** 1 Rudolf Virchow Center for Integrative and Translational Bioimaging, Julius-Maximilians-Universität Würzburg (JMU), Würzburg, Germany; 2 Department of Internal Medicine I, University Hospital Würzburg, Würzburg, Germany; 3 Comprehensive Heart Failure Center, University Hospital Würzburg, Würzburg, Germany; 4 Institute of Pharmacology and Toxicology, Julius-Maximilians-Universität Würzburg (JMU), Würzburg, Germany; 5 Department of Clinical Research & Epidemiology, Comprehensive Heart Failure Center, University Hospital Würzburg, Würzburg, Germany; 6 Institute of Clinical Epidemiology and Biometry, Julius-Maximilians-Universität Würzburg (JMU), Würzburg, Germany; 7 Institute for medical Data Science, University Hospital Würzburg, Würzburg, Germany; 8 Medical Clinic I, Cardiology and Angiology, Justus-Liebig-University Giessen, Giessen, Germany

**Keywords:** antibodies, anti-myocardial, human iPSC-CMs, immunofluorescence, myocardial infarction, myocarditis

## Abstract

Adaptive immune responses, particularly the production of anti-myocardial antibodies, have been implicated as critical mediators in myocardial healing and remodeling following myocardial infarction (MI), acute myocarditis (Myo), and heart failure (HF). However, current methods for detecting heart-reactive antibodies in patients are insufficient, as most rely on heart tissue slices from primates or other species. These approaches pose technical, logistical, and ethical challenges, including the risk of false negative results due to lack of inter-species cross-reactivity. To address these limitations, we developed a human cell-based assay to screen for patient-derived serum or plasma reactivity against human cardiomyocytes differentiated from induced pluripotent stem cells (iPSC-CMs). This human-on-human test system can detect cardiomyocyte-specific immunoglobulins present in patient plasma by applying indirect immunofluorescence staining, followed by convenient visualization through either confocal microscopy or standard widefield systems available in clinical laboratories. Overall, this approach provides a physiologically relevant and ethically responsible model for rapid, accurate testing of anti-myocardial antibodies across various clinical settings, thus offering accessible tools to stratify patients with myocardial disease according to their adaptive immune response status.

## 1 Introduction

Cardiac injury following myocardial infarction (MI), acute myocarditis (Myo), dilated cardiomyopathy (DCM), or acute heart failure (HF) decompensation can trigger adaptive immune responses directed against myocardial antigens released by dying cardiomyocytes (1–3). Specific T cells and autoantibodies targeting cardiac antigens, like myosin heavy chain, troponin, and beta 1-adrenergic receptors, have been reported in a broad range of conditions in both mice and patients (4–8). The prevalence of these autoantibodies is stated to be approximately 30% (9, 10). In particular, previous clinical studies have reported that the presence of heart-reactive antibodies associates with worse outcomes, after MI or acute HF decompensations, including increased mortality and a higher risk of rehospitalization (9–11). Likewise, preclinical studies have shown that immunoglobulin-deficient and B cell-depleted mice exhibit blunted cardiac adverse remodeling following experimental MI (12, 13).

Growing evidence that antigen-specific immune responses can influence individual myocardial disease progression has sparked interest in developing immune-based diagnostic tools to stratify patients according to their inflammatory burden, as well as in designing interventional immunomodulatory strategies to blunt pathogenic immune responses directed against cardiac antigens. The ongoing RITA-MI trial, for instance, is investigating whether B-cell depletion using an anti-CD20 antibody (Rituximab) can effectively limit B-cell responses following MI and thereby improve myocardial disease outcomes (14). On the diagnostic front, while previous studies have monitored the presence of serum antibodies specific to defined cardiac antigens (e.g., myosin and troponin), broader antigen-agnostic screening of anti-myocardial antibody reactivity has been hindered by the lack of appropriate tools.

Considering the obvious challenges of testing patients’ serum reactivity against human heart samples, a commonly utilized alternative for detecting heart-reactive antibodies in patients with myocardial diseases involves employing primate heart tissue sections as a substrate to identify antibody binding patterns associated with autoimmunity (9, 15). However, testing human antibodies on tissues obtained from other species can yield false-negative results due to the lack of cross-reactivities between species, potentially compromising the relevance of observations. Moreover, that such approaches require mammalian heart specimens pose challenges under current animal welfare standards. In addition, sample accessibility and logistical issues can limit the widespread clinical implementation of these antibody screening methods. These limitations highlight the need for alternative approaches that align with the 3Rs principles (Replacement, Reduction, and Refinement), which aim to minimize animal use in research while encouraging the development of ethical, accurate, and reproducible alternatives, including assessing anti-myocardial antibody reactivities in well-defined conditions (16).

Cell culture-based binding assays are already widely applied in other clinical settings to detect autoantibodies in various disease contexts, such as polyneuropathy (17), autoimmune thrombocytopenia (18), and rheumatic and connective tissue diseases (19). In 1975, antinuclear antibody detection in connective tissue disease shifted from tissue sections to cell culture-based assays to achieve higher sensitivity (20). Given the high reliability of induced pluripotent stem cell (iPSC) technology, which enables differentiation of human cardiomyocytes (21, 22), and the convenient access to fluorescence imaging (23) in most modern clinical medicine facilities, we developed a human-on-human indirect immunofluorescence assay to detect patients’ serum reactivity against human iPSC-derived cardiomyocytes (iPSC-CMs). Using standard and confocal fluorescence microscopy, we test human antibodies from patient serum on human cardiomyocytes and visualize antibody binding within cardiac cells, and the assay results achieve high spatial and cellular resolution while eliminating the need for animal experiments by testing antibody reactivity in the same host species.

## 2 Methods

### 2.1 Sample collection

For indirect immunofluorescence detection of autoantibodies in the plasma of patients with MI, the ANTIK-MI (Antikörper-Produktion durch B-Zellen nach Myokardinfarkt) study was conducted at the University Hospital Würzburg (reference: 240/20-am). A serum sample from a patient diagnosed with Myo was taken from the ‘Immunopathological pathways underlying myocarditis and inflammatory cardiomyopathy—an exploratory study (ImmpathCarditis)’, Cantonal Ethics Committee Zurich, permission 2021–01917 study. We thank Burkhard Ludewig for providing access to the sample. Samples from healthy subjects were collected from the population-based STAAB cohort study (vote #98/13), comprising a representative sample of residents of the city of Würzburg (Germany) without self-reported heart failure and aged 30–79 years at inclusion (24). All these studies were approved by local ethic boards and met all criteria of the Declaration of Helsinki. All patients provided informed consent.

### 2.2 Cell culture and differentiation

iPSC from two healthy donors without any documented cardiovascular diseases were cultured and differentiated into ventricular iPSC-CMs as described previously (25) via sequentially targeting the Wnt pathway (26). In brief, iPSCs were cultured in a monolayer on Geltrex^®^-coated dishes in chemically defined E8 medium (Life Technologies) until 80–90% confluency. To initiate cardiac differentiation Wnt signaling was activated by changing the medium to cardio differentiation medium (RPMI 1640 with GlutaMAX medium (Gibco) supplemented with 0.02% L-ascorbic acid 2-phosphate (Sigma-Aldrich) and 0.05% albumin (Sigma-Aldrich)), supplemented with the GSK3 inhibitor CHIR99021 (4 µmol/L, Millipore). 48 h later, Wnt signaling was inhibited by changing to cardio differentiation medium supplemented with 5 µmol/L of inhibitor of Wnt production-2 (IWP2, Millipore) for another 48 h. Subsequently, the medium was changed to cardio differentiation medium without the addition of small molecules for 48 hours. From day 6 onward, the medium was changed to cardio culture medium (RPMI 1640 with GlutaMAX medium supplemented with 2% B27 with insulin (Gibco)), which was changed every 2 to 3 days. Between days 14 and 18, cells started beating. Metabolic selection was used to purify the iPSC-CMs, and their purity was determined by Flow Analysis (>80% cardiac troponin T positive) and qPCR analysis for cardiac ventricular subtype markers (MLC2v vs. NR2F2). 7 days before fixation, 125,000 quality-proven ventricular iPSC-CM were seeded on glass coverslips (Epredia, CB00180RAC20MNZ0, #1.5, 18 mm) in a 12-well plate. iPSC generation was approved by the local ethics committee of the University of Goettingen (10/9/15) with the written consent of the donors. The study received proper ethical oversight (25).

Human Cardiac Myocytes (HCM, C-12810, PromoCell) were cultured in myocyte growth medium (C-22070, PromoCell) according to the manufacturer’s instructions in 37 °C with 5% CO_2_ in a humidified atmosphere. 2 days before fixation, the cells were seeded on glass coverslips (A. Hartenstein, DHR3, #1.5, 18 mm) in a 12-well plate.

HEK293T cells (ACC 872, Leibniz Institute DSMZ-German Collection of Microorganisms and Cell Cultures GmbH) were cultured in DMEM (41966-029, Gibco) supplemented with 10% Fetal bovine serum (FBS, A5256701, Gibco) and 1% Penicillin-Streptomycin (Pen-Strep, P4333-100ML, Sigma Aldrich) according to the manufacturer’s instructions in 37 °C with 5% CO_2_ in a humidified atmosphere. 24 hours before fixation, 75000 cells were seeded on glass coverslips (A. Hartenstein, DHR3, #1.5, 18 mm) covered with poly-D-lysine hydrobromide (PDL, P6407-5MG, Sigma-Aldrich) in a 12-well plate.

### 2.3 Indirect immunofluorescence

Human iPSC-CMs, HCMs, or HEK293T cells were fixed with 4% formaldehyde (FA, 252549-100ML, Sigma-Aldrich) for 20 minutes at room temperature. The cells were permeabilized with 0.1% Triton X 100 (3051.2, Carl Roth) in phosphate-buffered saline (PBS) for 5 minutes and blocked with 0.1% Triton X 100 in PBS with 1% bovine serum albumin (BSA, 8076.2, Carl Roth) for at least 1 hour. After the pre-treatment, the cells were incubated with patient or healthy control plasma or serum in the respective dilution in 1% BSA in PBS with 0.1% Triton X 100 (1:10, 1:50, or 1:100). Next, the cells were stained with mouse anti-alpha actinin (A7811-.2ML, Sigma-Aldrich, 1:500) or anti-β-tubulin (T8328, Sigma-Aldrich, 1:200). Goat anti-human IgG Alexa Fluor 555 (A-21433, Thermo Fisher Scientific, 1:200) and goat anti-mouse IgG Alexa Fluor 488 (A-11001, Thermo Fisher Scientific, 1:200) secondary antibodies were used. Stainings were performed in a moisture chamber by flipping the coverslips on top of a drop of staining solution first with primary antibodies (serum and anti-alpha actinin) and then with secondary antibodies (anti-human IgG and anti-mouse IgG) each for 1 hour at room temperature in the dark. Before mounting the sample on a glass slide with mowiol (Mowiol^®^ 4-88, 0713.1, Carl Roth), the cells were washed with PBS three times for 5 minutes at room temperature. During the last washing step, Hoechst (bisBenzimid H 33342, B2261, Sigma, 1:3000) was added for nucleus staining.

### 2.4 Image acquisition and analysis

Fluorescence imaging was performed using a laser scanning confocal microscope (LSM 980 Airyscan 2, Zeiss, Oberkochen, Germany, objective: 63x oil immersion, NA 1.4) or a widefield fluorescence microscope (DMI6000, Leica Microsystems, Wetzlar, Germany) equipped with either a 20x (air, NA 0.7) or a 63x (oil immersion, NA 1.4) objective. Confocal multichannel images were deconvolved using Huygens Professional (Scientific Volume Imaging, Hilversum, The Netherlands). The processed confocal or raw widefield images were then imported into the open-source image analysis software Fiji (27) for contrast adjustment and saved in PNG format for visualization.

The mean fluorescence intensity was analyzed using Fiji, with the cell area selected as the region of interest for serum and plasma intensity measurements, and the nucleus area for nucleus intensity (normalized by maximum intensity of the patient confocal images or mean intensity of the control widefield images). Statistical analysis was performed on 10 or 50 cells per condition, using one-way ANOVA with Tukey *post-hoc* test (p = 0.05) with OriginPro 2023b (OriginLab Corporation, Northampton, MA, USA).

## 3 Results

### 3.1 Anti-myocardial autoantibodies bind to iPSC-CMs

**Figure 1:**
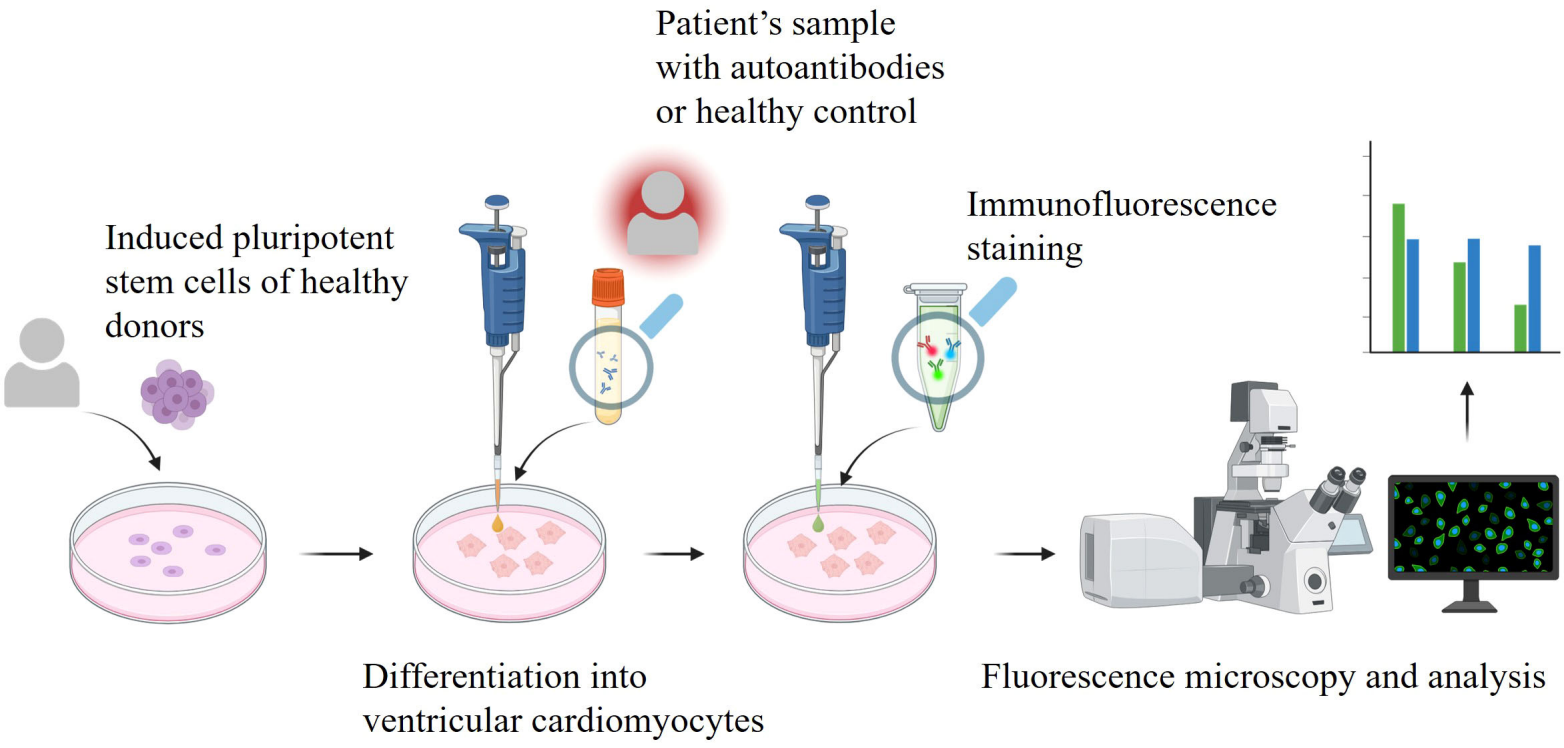
Schematic representation of the human-iPSC-CM-derived immunofluorescence assay workflow. Induced pluripotent stem cells of healthy donors are differentiated into cardiomyocytes. Patient’s or healthy control samples are added to the cells together with secondary antibodies for immunofluorescence staining. Fluorescence microscopy is performed, and the acquired images are quantitatively analyzed. Created in Biorender.com.

We used indirect immunofluorescence to explore whether antibodies present in the serum of patients diagnosed with MI or Myo exhibit binding to human iPSC-CM. A schematic of our established human-on-human indirect immunofluorescence assay to assess antibody binding is shown in **Figure 1**. Conventional immunocytochemistry protocols (28) typically involve culturing cells in well chambers and incubating the primary antibody overnight at 4 °C. To prevent evaporation, these protocols require the cells to be completely covered with staining solution, often necessitating large volumes of antibody. This is an impractical and potentially cost-prohibitive requirement in clinical settings, where patient biomaterial is limited. Our protocol instead utilizes a moisture chamber setup in which coverslips with adherent cells are inverted onto small drops of staining solution. The moisture chamber consists of a Petri dish lined with wet paper towels, thus reducing the required antibody volume from approximately 300 µl (in a 12-well plate) to just 30 µl. To further streamline the procedure, we performed simultaneous staining with both primary antibodies (anti-α-actinin and human sample with autoantibodies) and both corresponding secondary antibodies (anti-mouse IgG and anti-human IgG). The standard blocking and staining buffer was not further optimized, as it already yielded minimal nonspecific staining.

**Figure 2:**
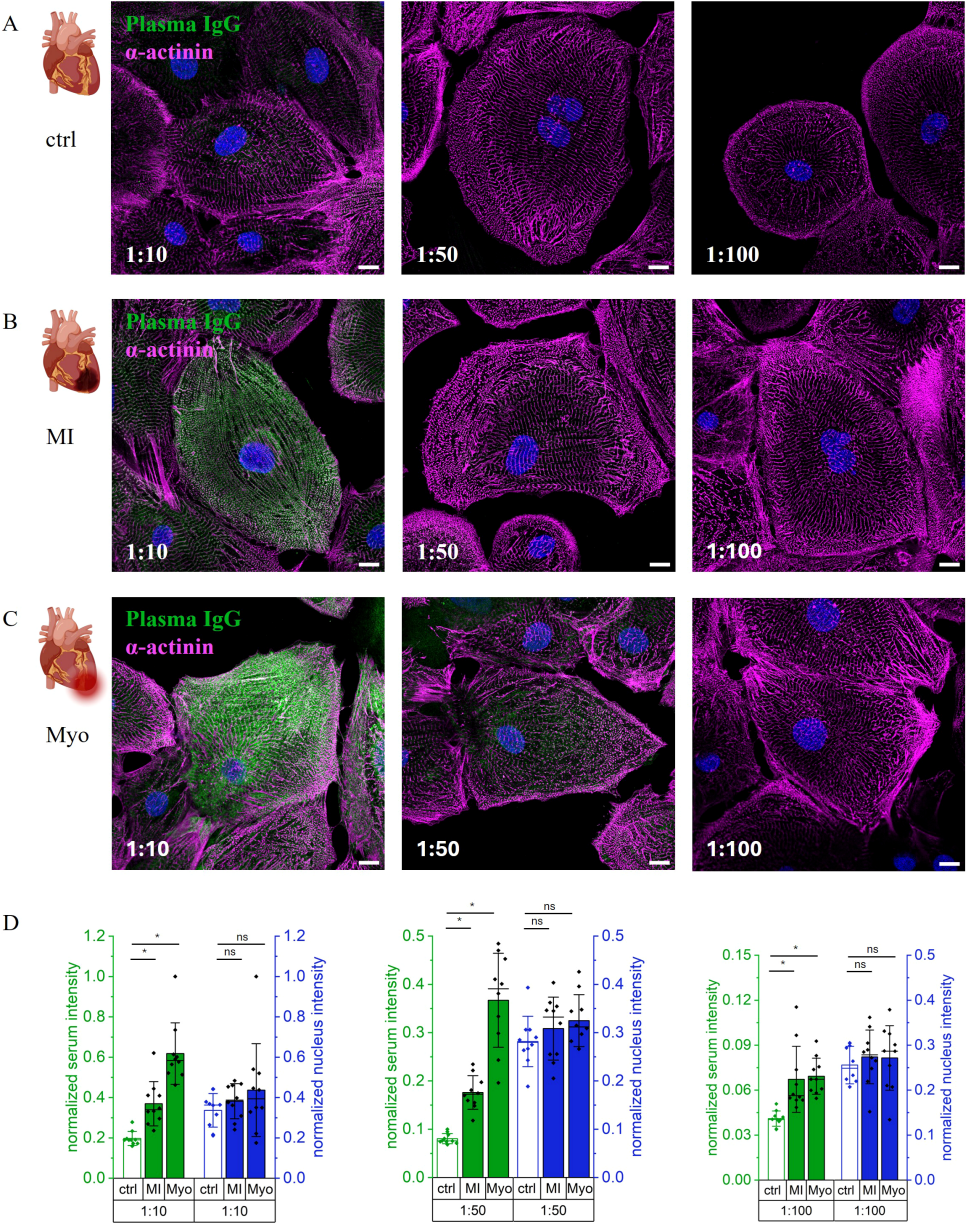
Dose dependent binding of Patients serum to human derived cardiomyocytes at day 90 after start of differentiation. **(A)** Serum of healthy control binding to cardiomyocytes with 1:10, 1:50, and 1:100 dilution. **(B)** Serum of patient after myocardial infarction with 1:10, 1:50 and 1:100 dilution. **(C)** Serum of Myocarditis patient with 1:10, 1:50, and 1:100 dilution. **(A-C)** Serum (anti-human IgG A555) in green, alpha-actinin (anti-mouse IgG A488) in magenta, and Hoechst staining for nucleus in blue. Scale 10 µm. **(D)** Normalized intensity for serum (green) and nucleus (blue) for healthy control (ctrl) derived from **(A)**, MI derived from **(B)**, and Myo derived from **(C)** (mean intensity per cell). Data is represented as bar graph with mean +/- SD. *p < 0.05 significant differences by one-way Anova with Tukey *post-hoc* test. Pictograms from BioRender.com.

With minimized volume of human sample required per staining, immunostaining was carried out on iPSC-CMs, 90 days post differentiation initiation. Prior to fixation and staining, cell purity and quality were evaluated by flow cytometry, which confirmed that over 80% of the population expressed cardiac troponin. Only ventricular cardiomyocytes were included in the experiments, and their phenotype was validated by quantitative PCR (qPCR) analysis. Staining with anti-α-actinin, a well-established cardiomyocyte marker (29), revealed the characteristic sarcomere stripe pattern in the confocal images (**Figures 2A–C**).

To identify optimal antibody concentrations, we tested different serum dilutions of patients (**Figures 2B, C**) and healthy control samples (**Figure 2A**). Among the tested conditions, the 1:10 dilution produced the most pronounced qualitative difference in fluorescence intensity (**Figure 2D**). For quantitative assessment, the mean fluorescence intensity of 10 individual cells was measured and compared. Across all tested dilutions, patient-derived samples (MI and Myo) exhibited significantly higher fluorescence signals than the healthy control (p < 0.05) (**Figure 2D**, green), while the nuclear signal remained constant, as expected (**Figure 2D**, blue). In all groups (MI, Myo, and healthy control), the serum-derived signal (**Supplementary Figure 1**) significantly decreased from 1:10 to 1:100 dilution (p < 0.05), a result that confirms the concentration-dependent nature of the observed staining.

### 3.2 Simplified imaging pipeline for clinical settings

**Figure 3:**
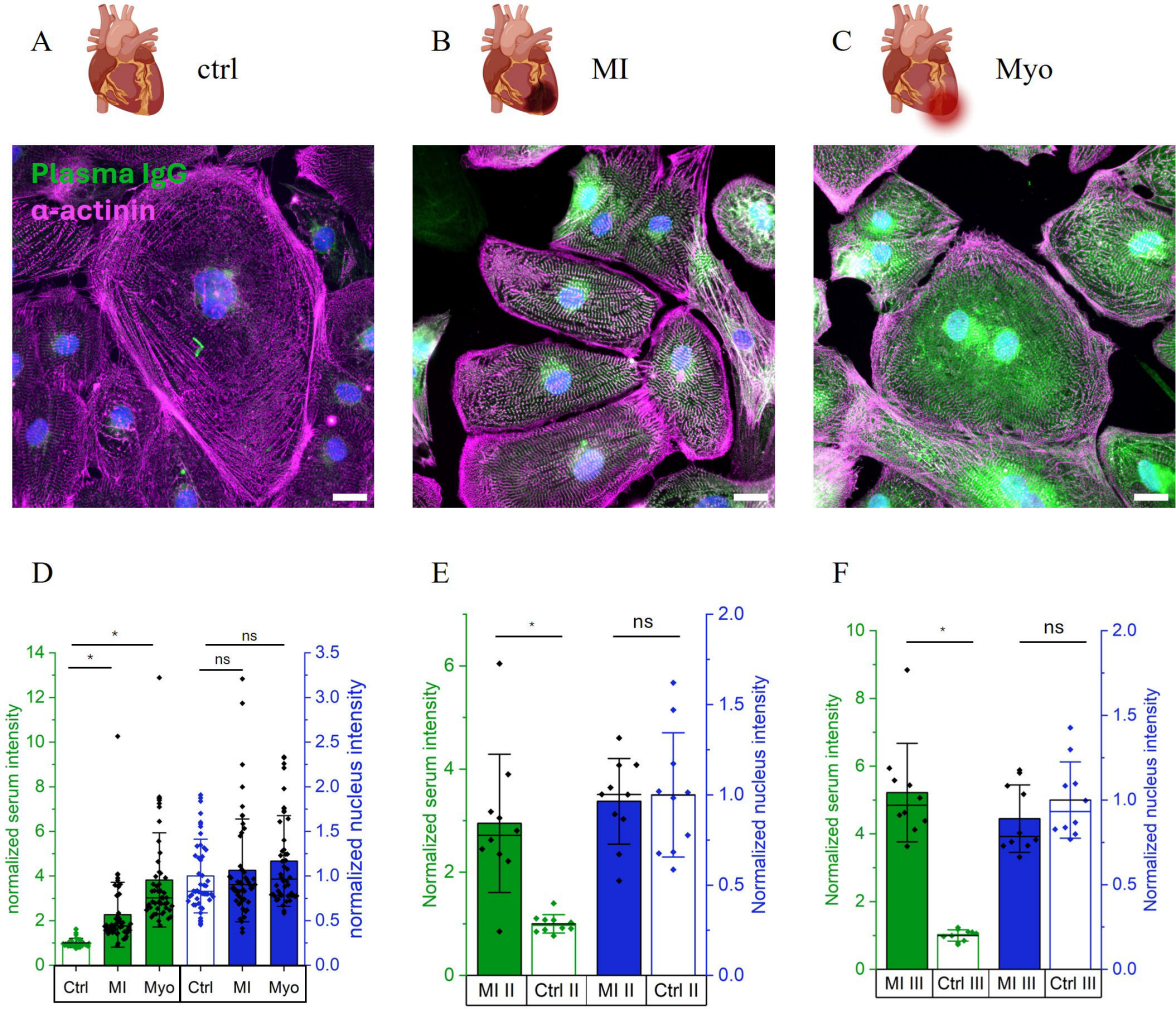
Translation to low expert labs with widefield fluorescence microscopy. **(A)** Widefield fluorescence image of 1:10 diluted serum of myocardial infarction patient. B) Widefield fluorescence image of 1:10 diluted serum of myocarditis patient. C) Widefield fluorescence image of 1:10 diluted serum of healthy donor. **(A-C)** Serum (anti-human IgG A555) in green, alpha-actinin (anti-mouse IgG A488) in magenta, and Hoechst staining for nucleus in blue. Scale: 20 µm. **(D)** Normalized intensity for serum (green) and nucleus (blue) for MI derived from **(A)**, Myo derived from **(B)**, and healthy control (ctrl) derived from **(C)** (mean intensity per cell). Data is represented as bar graph with mean +/- SD. *p < 0.05 significant differences by one-way Anova with Tukey *post-hoc* test. **(E, F)** Normalized intensity for serum (green) and nucleus (blue) for MI and healthy control (ctrl) from two independent experiments. Pictograms from BioRender.com.

Next, we sought to demonstrate that the 1:10 diluted samples from **Figure 2** can be effectively imaged using conventional widefield fluorescence microscopy, which is a more accessible and cost-efficient modality for clinical environments. To facilitate our protocol’s into clinical practice, particularly for high-throughput applications, it was essential to optimize not only the staining protocol but also the imaging and quantification steps. By combining conventional widefield fluorescence microscopy with open-source software for intensity analysis, we established a streamlined pipeline suitable for screening patients for heart-reactive antibodies. **Figures 3A–C** shows widefield fluorescence images of human iPSC-CMs previously analyzed by confocal microscopy (**Figure 2**). While the widefield images exhibit reduced contrast compared to confocal counterparts, the characteristic sarcomere stripe pattern remains discernible, thereby allowing reliable identification of cardiomyocytes. As observed in the confocal data, fluorescence intensity was markedly higher in samples from MI (**Figure 3B**) and Myo patients (**Figure 3C**) compared to the healthy control (**Figure 3A**). Quantifying the mean intensity from 50 representative cells confirmed these findings (**Figure 3D** in green), with significantly elevated signals in patient samples (p < 0.05), while nuclear staining remained unchanged (**Figure 3D**, blue). Analysis of the MI and healthy control samples in independent experiments demonstrate the robustness of our assay. **Figures 3E, F** show the quantitative comparison of two experiments distinct from the data shown in **Figure 3D**. The mean intensity of the MI II and the MI III samples is significantly higher than Ctrl II and Ctrl III.

**Figure 4:**
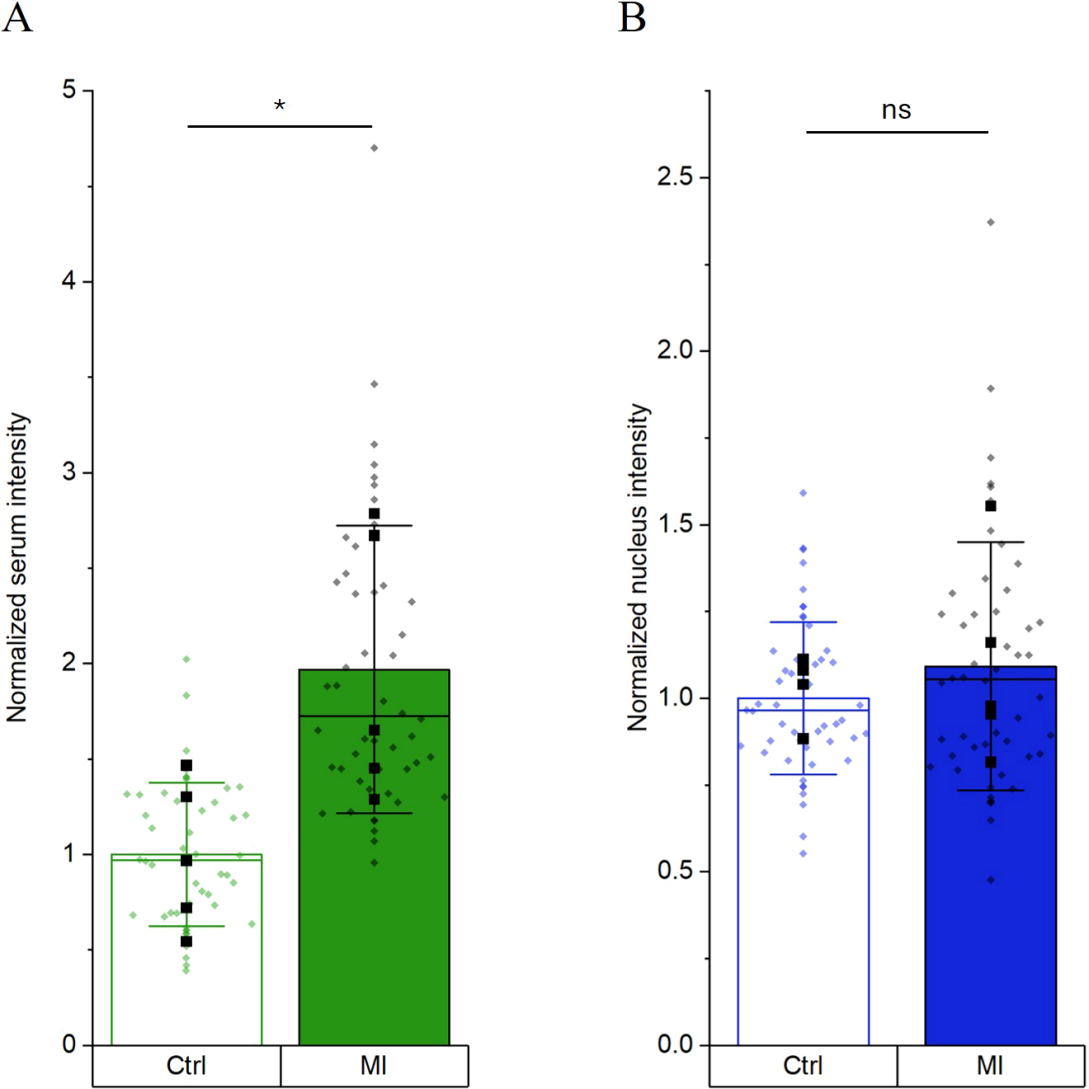
Comparison of five patients per group. Normalized intensity for serum **(A)** and nucleus **(B)** of 10 cells from 5 different patients per group (MI) in comparison to the healthy control (Ctrl). Mean of each patient and mean of each healthy control represented as black square. Data is represented as bar graph with mean +/- SD. *p < 0.05 significant differences by one-way Anova with Tukey *post-hoc* test.

In **Figure 4**, five patients per group are compared. The mean intensity of the patient cohort is significantly higher than the mean intensity of the control cohort (**Figure 4A**) while the nucleus signal remains constant (**Figure 4B**). The mean values for the single patients per group are represented by black squares symbols. The data in **Figure 4** additionally highlights the diversity of antibody binding in patients and control samples since different patients and controls exhibit different normalized mean intensities. Representative images of the five patients and control samples are shown in **Supplementary Figure 2**.

To ensure that the observed autoantibody signal truly reflects autoantibody binding in the patients samples, we included a quantitative secondary-only measurement where no primary antibody (anti-alpha actinin or patient/healthy control serum) is added to the iPSC-CMs. As seen in **Supplementary Figure 3A**, only the nuclei are visible. Analysis of MI sample and healthy control reveal higher signals than the secondary-only sample (**Supplementary Figure 3B**) while the nucleus signal remains constant (**Supplementary Figure 3C**), a strong hint that our assay represents true patient autoantibody binding.

Given the time-sensitive nature of diagnostics in clinical settings, we explored whether earlier time points in iPSC-CM maturation could still yield reliable results. As shown in **Supplementary Figure 4**, cardiomyocytes at day 60 post differentiation already showed clear and significant differences in signal intensity between patient and control sera across all tested dilutions (1:10, 1:50, and 1:100), similar to those observed at day 90. To further reduce the experimental timeline, we explored cardiomyocytes at day 20 post differentiation. As seen in **Supplementary Figure 5**, the signal of the MI sample is qualitatively and quantitatively higher than the signal of the healthy control. It is important to note that cells in an early differentiation status are smaller with rounder shape (seen in **Supplementary Figures 5A, B**) than the fully differentiated cells at day 90 or 60 (see **Figure 2**; **Supplementary Figure 4**).

For laboratories where no cardiomyocytes from iPSCs are available, using a commercial cell line might be an alternative. Thus, we tested the commercial primary human cardiac myocytes cell line which was not suitable for our purposes due to their immature phenotype and progenitor nature. (see **Supplementary Figures 6**, **7** illustrating the lack of the mature sarcomere stripe pattern typically observed in iPSC-CMs without consistent autoantibody binding for the control and the MI sample).

As a non-cardiac cell control, we used HEK293T cells as the substrate for antibody binding (see **Supplementary Figure 8**), where the binding is positive for the MI patient serum, showing lower signal for the healthy control as expected in the presence of endogenous β-adrenergic receptors.

## 4 Discussion

In this study, we developed and validated a workflow, based on indirect immunofluorescence of human iPSC-CM cultures, to screen for heart-reactive antibodies in the plasma of patients with MI and Myo. The assay herein reported directly addresses a long-standing gap in immune-based diagnostics in cardiology: the lack of validated human-on-human test systems to detect heart-reactive antibodies in patients with myocardial diseases. So far, systematically testing human serum antibodies on human myocardial tissue has been hindered by negligible sample availability and evident ethical and logistical constraints. Moreover, testing human serum antibodies on human hearts would be challenging because of endogenous immunoglobulins already present in the tissue. Macaque heart slices have been a well-established alternative to screen patient serum antibodies reactive to cardiac antigens (9). While this approach provides a feasible option, it relies on inter-species cross-reactivity and is therefore prone to false-negative results. Moreover, this method still requires primate experimentation, which can now be avoided, refined, and replaced. In light of these limitations, various groups have also tested serum reactivity directed against candidate antigens, such as myosin, troponin, and beta-1 adrenergic receptors (6, 7, 30). While these targeted approaches offer reliable means to detect specific antibody reactivities, they can only distinguish a limited number of antigens defined *a priori*. However, considering the great heterogeneity among myocardial disease patients, who have a broad range of comorbidities, risk factors, and HLA genetic diversity, approaches that rely on recognizing a small set of pre-defined antigens may be insufficient to capture patient heterogeneity. By leveraging iPSC technologies, our study addresses these gaps by validating a human-on-human assay that enables the unbiased detection of cardiomyocyte-reactive antibodies.

In this context, accumulating evidence highlights the clinical relevance of autoantibody screening in patients with heart disease, particularly regarding its prognostic value (31, 32). For instance, Baritussio et al. (32) demonstrated that adverse outcomes in myocarditis are closely associated with autoimmune features. These findings underscore the importance of reliably detecting autoantibodies in patient samples, as sensitive and specific assays may improve risk stratification and inform decisions on immunosuppressive therapy and follow-up strategies.

The use of cultured human cells in autoimmune diagnostics is well established and provides a conceptual framework for our approach. For example, HEp-2 cells have been employed since the 1970s for the detection of antinuclear antibodies, offering greater sensitivity than traditional tissue sections (20). Building on this paradigm, our human-to-human assay further extends the utility of cell-based diagnostics by employing iPSC-derived cardiomyocytes, thereby enabling clinically meaningful detection of anti-cardiac autoantibodies while facilitating standardization and scalability.

An additional advantage of iPSC-CMs is their capacity to be guided toward specific cardiac subtypes, such as atrial or ventricular phenotypes (33). This flexibility enables targeted investigation of antibodies relevant to specific clinical conditions. While our study focused on ventricular cardiomyocytes, future studies could adapt the protocol to examine atrial-specific antibodies, which are implicated in arrhythmogenic disorders (34, 35). Furthermore, using patient-derived iPSCs offers the opportunity to model genetic cardiomyopathies *in vitro* (29), an option that is particularly relevant given that sarcomere proteins, which are frequently mutated in these diseases, are established autoantibody targets (1, 36).

Importantly, our approach can be easily adjusted for the species of interest while remaining compliant with the principles of reduction, refinement, and replacement. Our method is compatible with standard widefield fluorescence microscopy, a system widely available in clinical laboratories (9, 37). Image acquisition is straightforward, and quantitative analysis can be performed using open-source software such as Fiji. Both confocal and widefield microscopy confirmed significantly increased signal intensities in patient samples, thereby validating the robustness and accessibility of the approach. The analysis of independent experiments further underscores the robustness of the assay where we see the same significant increase signal intensity in the patient sample.

Looking ahead, the integration of automation and AI-based image analysis (38, 39), has the potential to translate this platform into a clinically applicable diagnostics tool enabling high-throughput screening with improved reproducibility and reduced observer-dependent variability. In this setting, adapting the assay to a 96-well format would facilitate standardized workflows compatible with routine laboratory diagnostics and larger patient cohorts. Importantly, the use of Mowiol mounting provides a distinct clinical advantage by enabling long-term preservation of samples. This allows for retrospective analyses and direct comparison with newly collected patient material, supporting longitudinal monitoring, cohort-based studies, and potentially the evaluation of treatment responses over time.

Despite iPSC-CMs representing more of a fetal to neonatal stage of development and do not correspond to the maturity level of adult CM, the cultivation time of iPSC-CM is an important factor in determining their maturity level. Although long-term culture is typically employed to achieve cellular maturity (40), adopting specialized maturation media and forming 3D tissue-like structures can reduce the culture duration can reduce cell culture duration (41, 42), making the protocol more time-efficient and practical for clinical use, provided that a comparable differentiation state can be achieved. We were able to show that iPSC-CMs at different maturation stages (day 20, day 60, and day 90) are suitable for quantitative fluorescence antibody detection.

While iPSC-derived cardiomyocytes (iPSC-CMs) yielded robust and reproducible results, the use of commercially available cell lines could simplify the system, provided they adequately mimic mature cardiomyocytes. In our hands, the tested commercial cell line did not reproduce the findings obtained with iPSC-CMs. This emphasizes the importance of the cellular differentiation state. As shown in the supplementary data, the cell line displayed a different sarcomeric organization compared to differentiated cardiomyocytes, indicating relevant structural differences. Therefore, careful phenotypic characterization is essential to confirm successful differentiation (43) and to ensure assay reliability. Other commercial cell lines may still be suitable but require thorough validation.

Cryopreservation offers a practical solution for long-term storage and availability of cardiomyocytes. However, it has been reported to alter cellular and molecular properties. This requires careful validation and cautious interpretation when using cryopreserved cells in human-on-human indirect immunofluorescence assays (44).

Defining an appropriate non-cardiac control remains challenging. Many mammalian cells express β-adrenergic receptors (45, 46), which are targets of circulating autoantibodies in myocardial infarction (MI) (47, 48), heart failure (HF) (7, 49), and dilated cardiomyopathy (DCM) (50). Accordingly, HEK cells also show measurable antibody binding due to their intrinsic receptor expression.

Our assay is based on indirect immunofluorescence using total plasma, which contains a highly complex mixture of polyclonal antibodies. The human B cell repertoire is estimated to exceed 10¹² unique specificities (51). This differs fundamentally from assays using monoclonal antibodies. Binding observed across different cell types (e.g., cardiomyocytes and fibroblasts) does not necessarily indicate cross-reactivity of individual antibodies. Instead, it may reflect distinct antibody populations within the plasma. Therefore, this assay cannot distinguish true cross-reactivity from cumulative multi-reactivity at the clonal level.

Despite this limitation, indirect immunofluorescence assays using whole plasma remain widely used in immunodiagnostics and have proven clinical relevance. Examples include the detection of antinuclear antibodies in connective tissue diseases (20) and systemic lupus erythematosus (52), as well as anti-Caspr autoantibodies in polyneuropathy (17).

Use of cell fixation and permeabilization also warrants discussion. Fixation can mask or degrade certain epitopes, potentially reducing the efficiency of antibody binding (53). Permeabilization, although necessary to expose intracellular targets, may lead to protein loss, altered localization, and ultrastructural damage, thereby increasing the risk of false-positive results (54, 55). However, permeabilization, may also reveal otherwise inaccessible epitopes that could be particularly relevant for intracellular targets (54). For instance, β1-adrenergic receptors, a known target of autoantibodies in cardiac autoimmunity, are sensitive to fixation-related artifacts (1, 10, 56). Nonetheless, when carefully controlled, fixation enhances sample handling and enables delayed or repeated analysis. Moreover, autoantibodies targeting sarcomere structures, which are only accessible post-fixation, have been documented in several studies (1, 6, 30).

A final point concerns the use of healthy iPSC-CMs in our assay. MI results in extensive cardiomyocyte death and the release of damage-associated molecular patterns (DAMPs), which can trigger systemic autoimmunity (57). Consequently, assays using healthy cells may not capture some antibodies that develop in response to injury-specific antigens. However, given the clear signal differences observed between patient and control sera in our experiments, we believe this limitation is minor and can be addressed in future iterations by utilizing disease-modeling iPSC lines or alternative protocols.

In conclusion, we here report that employing iPSC-CM-based indirect immunofluorescence to screen for heart-reactive antibodies in patients with myocardial diseases provides not only a sensitive, reproducible, and suitable strategy for diagnostic purposes but also a promising alternative to traditional animal-based methods tissue-reliant assays. Our approach facilitates ethical, scalable, and human-relevant antibody detection in diseases such as myocardial infarction and myocarditis. Going forward, similar pipelines may be extended to identify autoantibodies associated with other cardiovascular or systemic autoimmune disorders. By presenting a convenient antibody screening pipeline compatible with large patient cohorts, our study could pave the way for future stratification of myocardial disease patients based on their individual immunoreactivity profiles. From a long-term perspective, immune-based diagnostics applied to cardiology could help establish objective criteria for identifying patients with residual inflammatory burden who may benefit from immunomodulatory interventions.

## Data Availability

The datasets presented in this study can be found in online repositories. The names of the repository/repositories and accession number(s) can be found below: Zenodo (DOI: 10.5281/zenodo.16983526).
