# Supplementary material for: A human-on-human assay for detecting anti-myocardial antibodies in patients with myocardial disease
Source: Front Immunol. 2026 May 8;17:1744039. doi: 10.3389/fimmu.2026.1744039 (PMC13194094; doi:10.3389/fimmu.2026.1744039)
Supplement: Supplementary file 1 [file DataSheet1.pdf]

Supplementary Material

1 Supplementary Figures

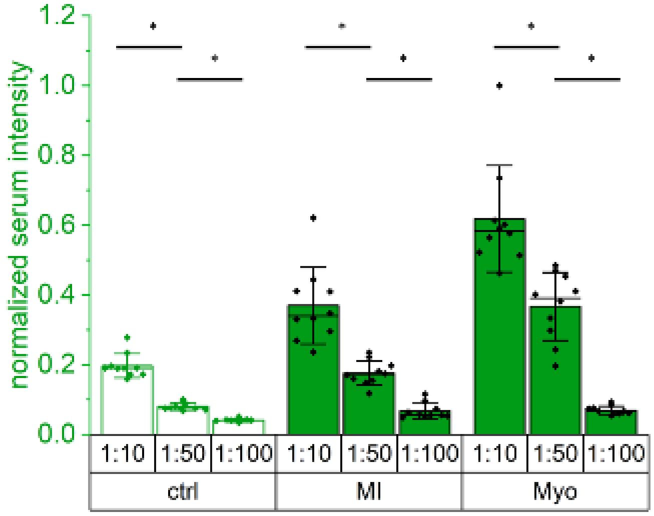

Supplement Figure 1: **Normalized fluorescence intensity of serum binding to human derived cardiomyocytes at day 60 after start of differentiation**. Intensities derived from Fig. 1 A-C (mean intensity per cell). Data is represented as bar graph with mean +/- SD. *p < 0.05 significant differences by one-way Anova with Tukey post-hoc test.

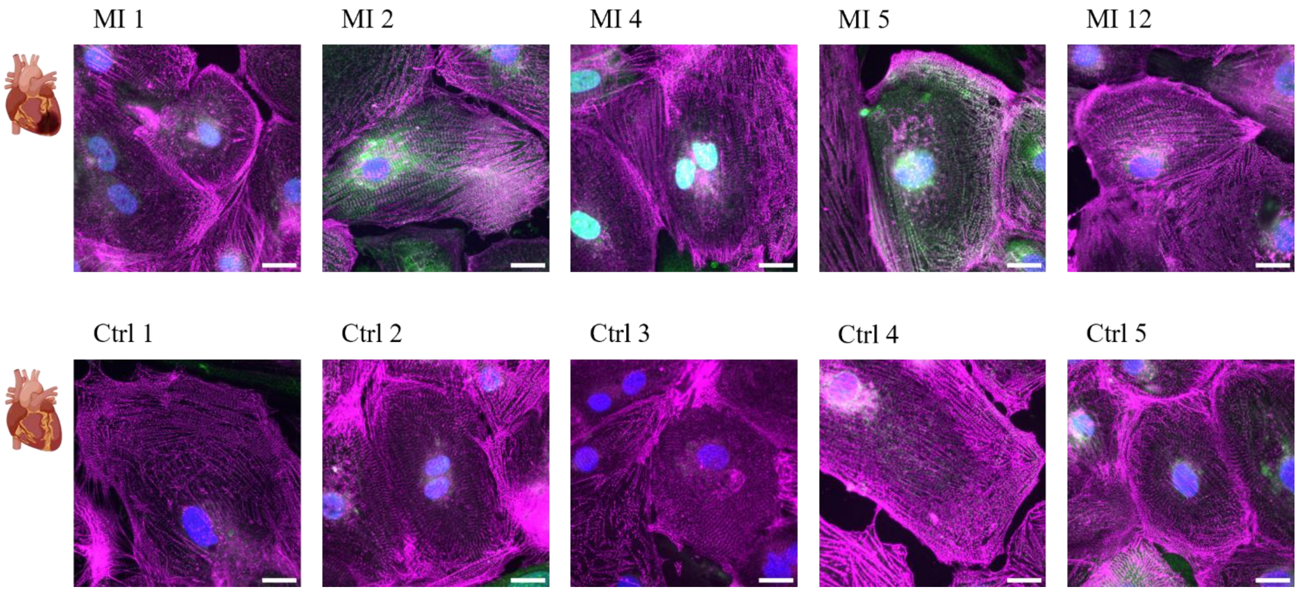

Supplementary Figure 2: **Comparison of five patients per group**. Representative images of five patients and control samples. Anti-human IgG A555 in green, Anti-mouse IgG A488 in magenta, and Hoechst staining for nucleus in blue. Scale 20 µm.

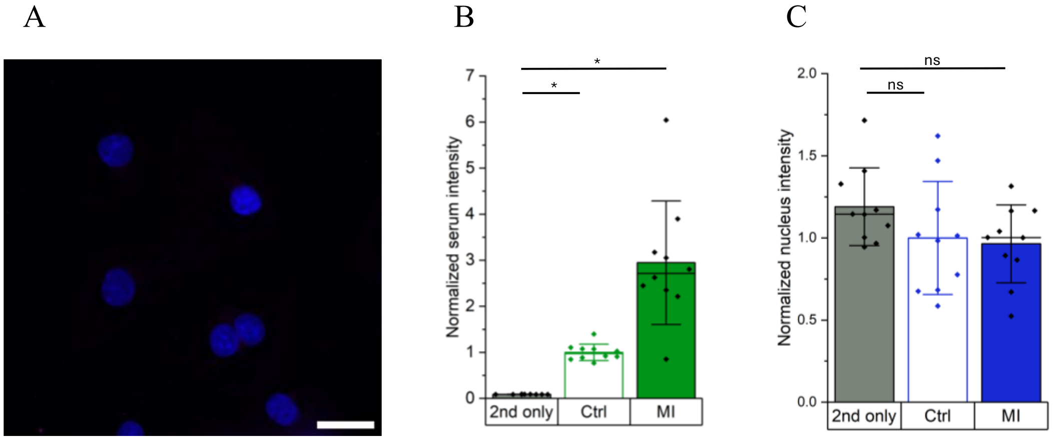

Supplementary Figure 3: **Secondary antibodies only control.** A) Representative image of iPSC-CMs stained only with secondary antibodies. Anti-human IgG A555 in green, Anti-mouse IgG A488 in magenta, and Hoechst staining for nucleus in blue. Scale 20 µm. B) Normalized intensity of serum from secondary only derived from A (grey), healthy control (white), and MI (green). C) Normalized intensity of nucleus from secondary only derived from A (grey), healthy control (white), and MI (blue) (mean intensity per cell). Data is represented as bar graph with mean +/- SD. *$p < 0.05$ significant differences by one-way Anova with Tukey post-hoc test.

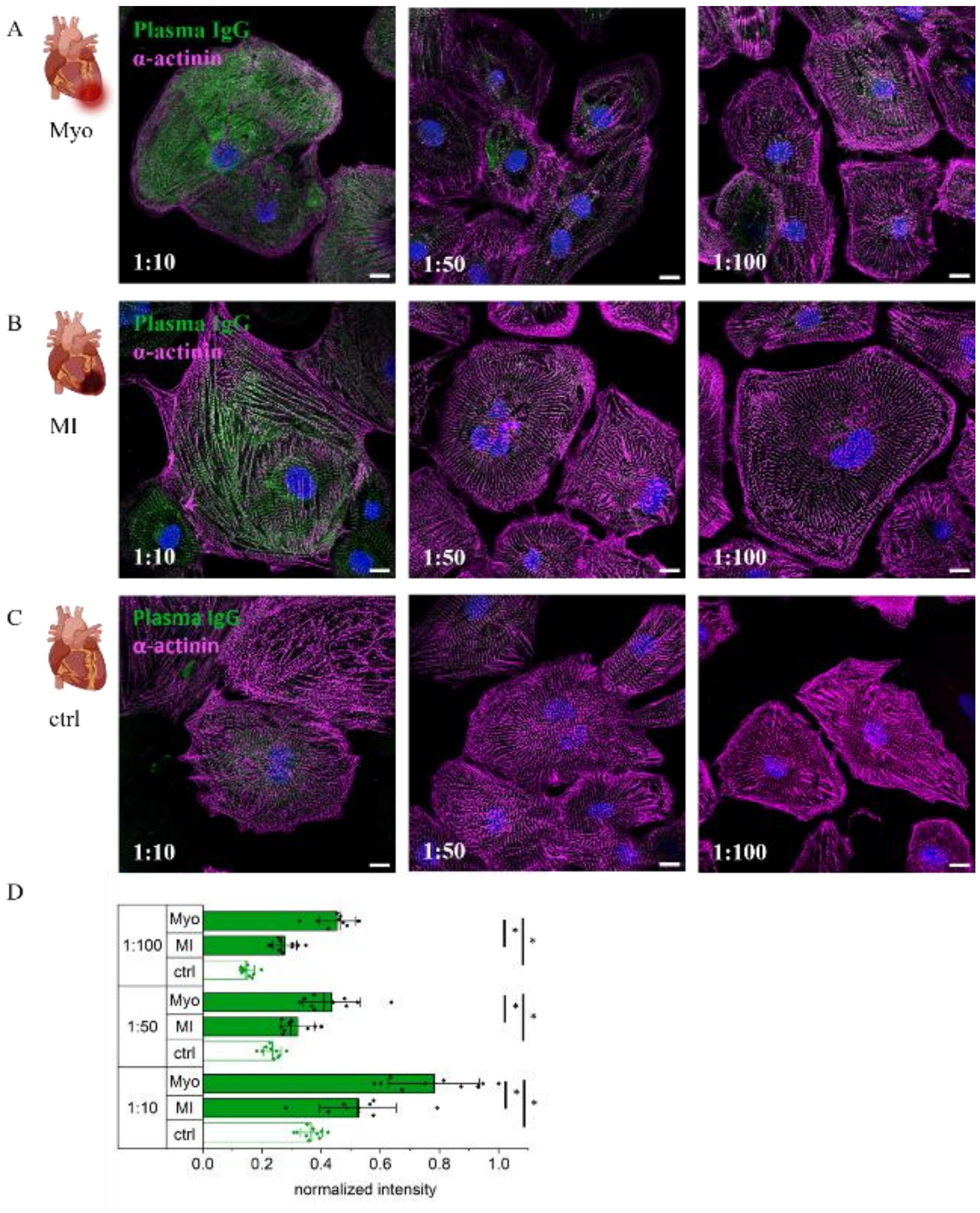

Supplement Figure 4: **Dose dependent binding of Patient serum to human derived cardiomyocytes at day 60 after start of differentiation.** A) Serum of Myocarditis patient with 1:10, 1:50, and 1:100 dilution. B) Serum of patient after myocardial infarction with 1:10, 1:50 and 1:100 dilution. C) Serum of healthy control binding to cardiomyocytes with 1:10, 1:50, and 1:100 dilution. (A-C) Serum (anti-human IgG A555) in green, alpha-actinin (anti-mouse IgG A488) in magenta, and Hoechst staining for nucleus in blue. Scale 10 µm. D) Normalized intensity for serum (green) and nucleus (blue) for Myo derived from A, MI derived from B, and healthy control derived from C (mean intensity per cell). Data is represented as bar graph with mean +/- SD. *$p < 0.05$ significant differences by one-way Anova with Tukey post-hoc test.

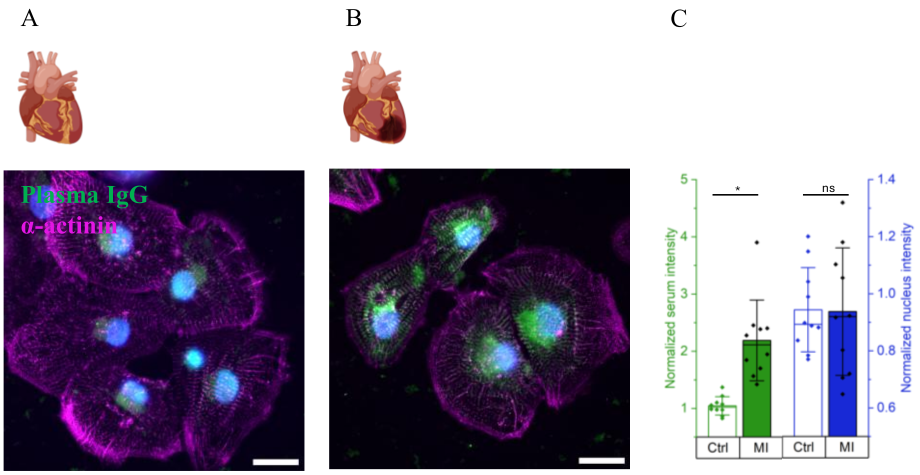

Supplementary Figure 5: **Immature cardiomyocytes.** A) Serum of healthy control binding to cardiomyocytes on day 20 after start of differentiation (d20). B) Serum of patient after myocardial infarction binding to d20 cardiomyocytes. (A-B) Serum (anti-human IgG A555) in green, alpha-actinin (anti-mouse IgG A488) in magenta, and Hoechst staining for nucleus in blue. Scale 20 µm. C) Normalized intensity for serum (green) and nucleus (blue) for healthy control derived from A and MI derived from B (mean intensity per cell). Data is represented as bar graph with mean +/- SD. *p <0.05 significant differences by one-way Anova with Tukey post-hoc test.

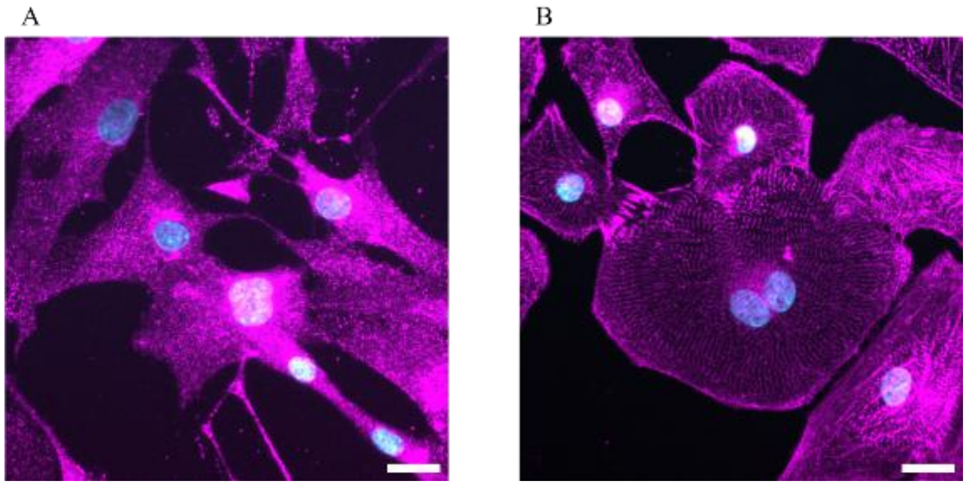

Supplement Figure 6: **Comparison of cytoskeleton of commercial human cardiac myocytes and induced pluripotent stem cell-derived cardio myocytes with widefield microscopy.** A) Human cardiac myocytes from PromoCell with anti alpha-actinin (magenta) and nucleus staining (Hoechst, cyan). B) Induced pluripotent stem cell-derived cardio myocytes with anti alpha-actinin (magenta) and nucleus staining (Hoechst, cyan). Scale: 20 µm.

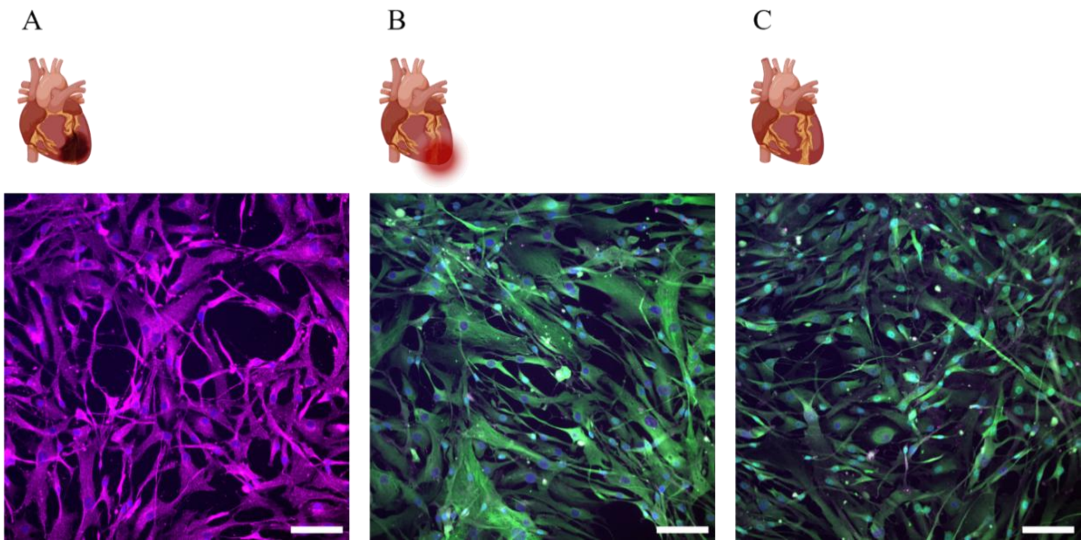

Supplementary Figure 7: **Human cardiac myocytes.** A) Serum of MI patient binding to Human cardiac myocytes. B) Serum of patient with myocarditis binding to Human cardiac myocytes. C) Serum of healthy control binding to Human cardiac myocytes. (A-C) Serum (anti-human IgG A555) in green, alpha-actinin (anti-mouse IgG A488) in magenta, and Hoechst staining for nucleus in blue. Scale 1 mm.

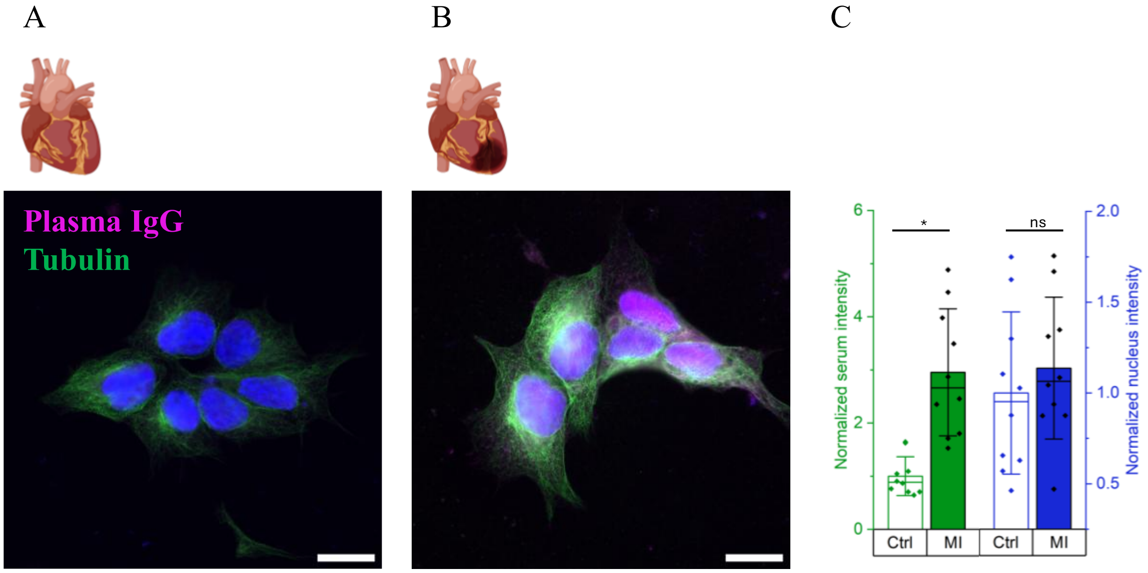

Supplementary Figure 8: **Non-cardiac cell control.** A) Serum of healthy control binding to HEK293T cells. B) Serum of patient after myocardial infarction binding to HEK293T cells. (A-B) Serum (anti-human IgG A555) in magenta, beta-tubulin (anti-mouse IgG A488) in green, and Hoechst staining for nucleus in blue. Scale 20 µm. C) Normalized intensity for serum (green) and nucleus (blue) for healthy control derived from A and MI derived from B (mean intensity per cell). Data is represented as bar graph with mean +/- SD. *$p < 0.05$ significant differences by one-way Anova with Tukey post-hoc test.
